# Supplementary material for: Needs of Older People Attending Day Care Centres in Poland
Source: Healthcare (Basel). 2020 Aug 29;8(3):310. doi: 10.3390/healthcare8030310 (PMC7551148; doi:10.3390/healthcare8030310)
Supplement: Supplementary file 1 [file healthcare-08-00310-s001.pdf]

Survey for the day care centres attendees in Poland

"Needs assessment of day care centres attendees in Poland"

Ladies and gentlemen,

We would like to ask you to fill in this survey.
Its aim is to assess the needs of the day care centres attendees on the territory of Poland.

In order to conduct the research, we would like you to answer the following questions.
The information obtained from you will enable us to get to know the needs of the day care centres attendees and prepare recommendations for creating a new offer in care centres.

The survey is anonymous, and all the obtained information will be used solely for the purposes of conducting a statistical analysis.

Thank you very much for the help and participation in the research!

DEMOGRAPHICS

Underline the correct answer.

1. **Sex**
   - ☐ 1. Woman
   - ☐ 2. Man

2. **Year of birth**

   - ☐ ……………..

3. **Educational background**
   - ☐ 1. Incomplete primary or primary education
   - ☐ 2. Vocational education
   - ☐ 3. Secondary education
   - ☐ 4. Higher education

4. **Previously practised profession**

   - ☐ …………………………………………

5. **Marital status**
   - ☐ 1. Single
   - ☐ 2. Married
   - ☐ 3. Widow / widower
   - ☐ 4. Divorced
   - ☐ 5. In an informal relationship
   - ☐ 6. Another term…………………

6. **Occupational situation**
   - ☐ 1. I am retired
   - ☐ 2. I'm on a disability pension
   - ☐ 3. Other ………………………………………………….

7. **Place of residence**
   - ☐ 1. City above 300 thousand inhabitants
   - ☐ 2. City between 100-300 thousand inhabitants
   - ☐ 3. City below 100 thousand inhabitants
   - ☐ 4. Village

8. **Time of attending the day care centre**
   - ☐ 1. over 5 years
   - ☐ 2. over 2 years
   - ☐ 2. 1-2 years
   - ☐ 3. Half a year - year
   - ☐ 4. Less than half a year

9. **How do you generally assess the availability of day care centres?**
   - ◻ 1. Very well
   - ◻ 2. Well
   - ◻ 2. Moderately
   - ◻ 3. Badly
   - ◻ 4. Very badly

Needs within the scope of the services of the day care centre

1. **Please put a cross "x" in the appropriate box that matches your assessment of the services provided in a day care centre.**

TYPE OF SERVICE	VERY WELL	WELL	NEITHER BADLY, NOR WELL	BADLY	VERY BADLY
WELFARE SERVICES **various forms of occupational therapy, music therapy, art therapy, memory trainings**					
WELFARE SERVICES **help in organising personal and administrative matters**					
**CARE AND HYGIENIC SERVICES related to, among other things, the possibility of using a shower, washing machine, monitoring blood pressure**					
SUPPORTIVE SERVICES **social gatherings, socialising events, leisure time and cultural activities**					
PREVENTIVE SERVICES **meeting health needs: educational classes, lectures given by invited guests**					
OTHER SERVICES **computer classes, foreign languages learning**					
**MEALS**					

2. Which services in the offer of the day care centre are the most important for you? Please select from the following ones and write in THREE most important services for you.

welfare // care // supportive // preventive // other

1…………………

2…………………

3…………………

**3. Which amidst the following WELFARE services in the offer of the day care centre are the most important for you?
Please select from the following ones and write in three most important WELFARE services for you.**

memory training // occupational therapy // meal //
help in organising personal and administrative matters

1…………………

2…………………

3…………………

**4. Which amidst the CARE services in the offer of the day care centre are the most important for you?
Please select from the following ones and write in three most important CARE services for you.**

using a shower // using a washing machine // blood pressure monitoring //
body mass measurement // nurse's care

1…………………

2…………………

3…………………

5. **Which amidst the PREVENTIVE actions in the offer of the day care centre are the most important for you?**
Please select from the following ones and write in three most important PREVENTIVE services for you.

lectures with doctors // lectures with the psychologist // lectures with the lawyer //
lectures with the social worker

1…………………

2…………………

3…………………

6. **Which amidst the SUPPORTIVE services in the offer of the day care centre are the most important for you?**
Please select from the following ones and write in three most important SUPPORTIVE services for you.

going to theatre // going to the cinema // going for a walk //
spending time together with other attendees of the day care centre and conversations

1…………………

2…………………

3…………………

7. **Which amidst OTHER services in the offer of the day care centre are the most important for you?**
Please select from the following ones and write in three most important OTHER services for you.

transportation guarantee // computer classes // access to a library

1…………………

2…………………

3…………………

**8. Which services are missing in the day care centre which you attend?
Please underline answers that are correct in your opinion.**

- ◻ 1. various forms of occupational therapy: music therapy, art therapy etc.
- ◻ 2. memory trainings
- ◻ 3. help in organising personal and administrative matters
- ◻ 4. meal
- ◻ 5. possibility of using the shower, washing machine
- ◻ 6. RR test, glucose testing, pulse test
- ◻ 7. social gatherings, socialising events
- ◻ 8. leisure time and cultural activities
- ◻ 9. educational classes, lectures given by invited guests, causeries with specialists
- ◻ 10. Other services are missing, which ones?
  ……………………………………………………………………………………………
- ◻ 11. No services are missing.

**9. Does any of the factors mentioned constitute a limitation for you to use the day care centre?
Please underline the answers that are correct in your opinion.**

- ◻ 1. Too long distance from home
- ◻ 2. Lack of suitable public transportation connections
- ◻ 3. Architectonic barriers (e.g. lack of a lift, lack of drive for the disabled)
- ◻ 4. Reluctance to leave home
- ◻ 5. Lack of time
- ◻ 6. Lack of financial resources
- ◻ 7. Lack of organised transport (e.g. bus transport)
- ◻ 8. Health condition
- ◻ 9. Unsuitable offer of activities offered in the day care centre
- ◻ 10. Other, which ones? …………………………………………………………………….
- ◻ 11. There are no factors limiting the use of the day care centre

**10. The current fee for attending the day care centre is for you:
Please underline the correct answer.**

- ◻ 1. Too high
- ◻ 2. Appropriate
- ◻ 3. I don't pay (I’m exempt from the fee)

11. Would you like to attend the following activities at the day care centres? Put a cross "x" in the appropriate box.

TYPE OF ACTIVITY	DEFINITELY YES	RATHER YES	HARD TO SAY	RATHER NOT	DEFINITELY NOT
**Learning foreign languages**					
**Art classes (e.g. painting, art, handicraft classes)**					
**Lectures on health situation**					
**Lectures on the legal situation of a senior**					
**Board games, card games**					
**Computer classes**					
**Photography workshops**					
**Movement classes (e.g. Nordic-walking poles, dance, aerobics)**					
**Cultural classes (e.g. book club, the cinema, the theatre)**					
**Trips**					
**Music classes**					
**Cooking classes (cooking)**					

12. Are there any classes that improve memory in the offer of your day care centre? Please underline the correct answer.

- ◻ 1. Yes
- ◻ 2. No

13. Do you have an opportunity to use a washing machine/bath/shower at the day care centre?
Please underline the correct answer.

- ◻ 1. Yes
- ◻ 2. No

14. Why do you attend the day care centre?

……

……

……

……

……

15. Please describe which needs an offer of the day care centre should meet.

……

……

……

……

16. How many persons, in your opinion, should simultaneously attend activities at the day care centre? Please write in the number of persons.

………………………………………………………………….

Housing situation

1. **I live:**
   Please underline the correct answer.

   - ◻ 1. Alone
   - ◻ 2. With my partner / wife / husband
   - ◻ 3. I live with my family (children, grandchildren)
     how many persons?..

2. **Are you satisfied with your housing situation?**
   Please underline the correct answer.

   - ◻ 1. Definitely glad
   - ◻ 2. Rather satisfied
   - ◻ 3. I don’t have an opinion
   - ◻ 4. Rather not satisfied
   - ◻ 5. Definitely not satisfied

Do you have any remarks/comments regarding the functioning of the day care centre?

....

....

....

....

One more time thank you very much for your help!

The survey is conducted within the framework of the project “Homely Marina” implemented in the area of Common Framework (contest no.: POWER.04.03.00-IP.07-00-004/16) co-financed with the resources of the European Social Fund within the framework of the Operational Programme Knowledge Education Development.
